# 

**Published:** 1827-07

**Authors:** 


### Contents

OF

### No. 13, Vol. VII. (NEW SERIES) for JULY, 1827.

[*THIRD NUMBER FOR* 1827.]

Art. I.

Mental Derangement.

1. M. Voisin on the Moral and Physical Causes of Mental Disorders, and of other Nervous Affections ........ } 1

2. Observations on the Causes, Symptoms, and Treatment of Insanity. By P. S. Knight M.D. ........ }

II.

Elements of Physics, or Natural Philosophy, General and Medical, &c. By N. Arnott, M.D ........ 35

III.

LEISURE HOURS IN PHYSIC AND SURGERY.

1. The Gold-headed Cane. By a Fellow of the Royal College of Physicians ........ } 38

2. Nugæ Canoræ; or Epitaphian Mementos of the Medici Family of Modern Times. By a Fellow of the College of Surgeons ........ }

IV.

Principles or Propositions of Medicine. By Professor Broussais. Part III. Therapeutics ........ 46

V.

TREATMENT OF URETHRAL STRICTURE.

1. Mr. Macilwain on Stricture of the Urethra ........ } 69
2. Mr. Andrews on Lunar Caustic in Strictures ........ }

VI.

ABDOMINAL DISEASES.

M.M. Andral and Lerminier on Diseases of the Abdominal Viscera, from the Clinique of La Charité, &c. ........ 73

VII.

Medical Botany. By Dr. Stephenson and Mr. Churchill ........ 95

VIII.

The Life of Edward Jenner, M.D. &c. By John Baron, M.D. &c. ........ 102

IX.

The Dublin Hospital Reports, and Communications in Medicine and Surgery. Vol. Fourth.

Art. I.—Clinical Observations. By Dr. Graves.

1. Abscess of the Liver, with a new Mode of opening the Abscess 112

2. Rheumatism of the Temporal Muscles, producing Lock-Jaw .. 113

3. Idiopathic Glossitis ........ 113

4. On Colica Pictonum ........ 114

5 & 6. On Disordered Intestinal Excretions ........ 115

7. Contagious Psoriasis ........ 116

8. On a peculiar Kind of Swelling of the Extremities ........ 116

II.—Case of remarkable Pulsation in the Veins. By Dr. Chas. Davis 118

III.—Dr. Crampton on Obstinate Constipation ........ 119

IV.—Dr. Cuming on a peculiar Affection of the Mouth in Children.. 120

V.—Mr. Speer's Case of Ruptured Cæcum ........ 121

VI.—Case of Cynanche Laryngea requiring Tracheotomy, and the continued Use of a Tube ........ 122

VII.—On an Ulcer of a peculiar Character ........ 123

VIII.—On fatal Erethism of the Stomach. By Dr. Cheyne ........ 124

### X.

### Quarterly Periscope

OF

### PRACTICAL MEDICINE.

### PART I.

1. Messrs. Bouillaud and Cruveilheir on the Anterior Lobes of the Brain, with Cases in Illustration.......... 129
2. Dr. Paul on the Effects of Kino in Dropsy .......... 134
3. Remarkable Case of double Hepatic Abscess .......... 135
4. Mr. Mackenzie on Catarrho-rheumatic Ophthalmia .......... 136
5. On the Effects of Alum in Malignant Croup .......... 142
6. Mr. Newnham on the Sedative Effects of Green Tea.......... 143
7. Case of severe Hæmaturia unexpectedly cured .......... 145
8. Fatal Case of Chorea, with the Dissection .......... 146
9. Mr. Arnott on the Nature and Contagious Character of Erysipelas.......... 148
10. The Proteian Malady—remarkable Case in the Person of a Medical Gentleman, related by himself.......... 149
11. Mr. Higginbottom on Lunar Caustic in various external Affections.......... 153
12. On a new Mode of Treatment in Cases of obstinate Chronic Diarrhœa.... 155
13. Aneurismal Tumour of the Knee, with unusual Symptoms. By Professor Lallemand .......... 156
14. Memoir on Sanguineous Tumours of equivocal Character, by Professor Breschet.......... 159
15. Dr. Cummin's Classification and Description of the various Forms of Mammary Disease.......... 162
16. Case of Chorea, cured by very large Doses of Arsenic .......... 164
17. M. Cruveilheir on Erosion and Destruction of Articulating Cartilages.... 165
18. Mr. Brodie on Poisonous Matter in Offal.......... 166
19. M. Broussais on Asthma .......... 167
20. Remarkable Cases of Abdominal Tumours .......... 169
21. Case of Poisoning by Arsenic and Laudanum.......... 170
22. Curious Case of Intermittent Rheumatic Fever.......... 171
23. M. Jacobson on the Nervous Communication between the Great Sympathetic and the Trifacial Nerve .......... 172
24. On the removal of Phthisical Patients to Warm Climates.......... 173
25. M. Broussais on Tetanus .......... 175

### PART II. HOSPITAL REPORTS.

1. M. Dupuytren's Case of Ligature of the Iliac Artery, attended with unusual circumstances—*(Hôtel Dieu.)* .......... 177
2. Sir Astley Cooper on Disease of the Nails—*(Guy's Hospital.)* .......... 181
3. M. Lisfranc on the Chlorides of Soda and Lime in Cases of Fistula—*(La Pitié.)*.......... 183
4. Dr. Mackintosh on Bleeding in the Cold Stage in Ague—*(Royal Ordnance Hospital.)* .......... 184
5. On Inflammation of the Gall-bladder, &c.—*(Hôpital Cochin.)* .......... 187
6. Mr. Travers on Wounded Arteries—*(St. Thomas's.)* .......... 188
   1. Ligature of the Carotid..........
   2. Subclavian Aneurism ..........
7. M. Alibert on the Morbus Pedicularis—*(St. Louis.)* .......... 189
   1. Psoris Papulosa ..........
   2. Psoris Crustacea .......... 191
8. On the Use of Balsam Copaiba and Cubebs in Injection—*(Hôpital de la Faculté.)*.......... 192
9. Messrs. Bell, Shaw, &c. on Injuries of the Head—*(Middlesex Hospital.)* .. 193
10. Quarterly Hospital Report—(Hôtel Dieu.)
   Intermittent Fevers .......... 195

Intermittent Nasal Hæmorrhage .......... 196

Remarkably obstinate Case of Singultus .......... 196

On Articular Rheumatism .......... 197

11. M. Broussais on Hypertrophy of the Heart—*(Val de Grace.)* .......... 199

12. Pathological Distinctions between the Nerves of Sense and Nerves of Motion, by Surgeon Broughton—*(Regimental Hospital, Dragoon Guards.)* .. 200

13. M. Velpeau on Puerperal Peritonitis—*(Hospice de Perfectionnement.)* .... 201

14. M. Dupuytren on the Operation for Cataract—*(Hôtel Dieu.)* .......... 208

15. Carotid Aneurism .......... 209

1. Mr. Wardrop's second Case .......... 209

Reasons for believing the Artery was not tied .......... 211

Criticisms on this Point of Surgery .......... 212

2. Mr. Lambert's Case of Carotid Aneurism .......... 214

Criticisms on Mr. Lambert's Case .......... 216

16. On Obstruction of Arteries, with several Cases in Illustration—*(La Pitié.)* 218

17. Amputation during the progress of Mortification—(*St. George's.*) ...... 219

18. Dr. Bouillaud on Hypertrophy of the Muscular Coat of the Stomach—(*Hôpital Cochin.*) .......... 221

19. Case in which Gastrotomy was performed for the Relief of Hernia. By Mr. Brodie—(*St. George's Hospital.*) .......... 223

PART III. ANALECTA.

1. On certain Periodical Diseases, without Fever. By Professor Fulci .... 225

*Case* 1. Periodical Gonorrhœa .......... 225

—— 2. Periodical Neuralgia .......... 225

—— 3. Periodical Insanity .......... 226

—— 4. Periodical Ascites .......... 227

—— 5. Periodical Neurosis .......... 227

2. M. Magendie on the Quantity, Use, and Quality of the Cephalo-Spinal Fluid, with Cases .......... 228

3. Mr. Earle's Case of Acute Tetanus, with the Appearances on Dissection .. 232

4. Dr. Elliotson's Case of Aortic Aueurism .......... 233

5. Two Cases of Poisoning by Belladonna .......... 233

6. M. Leroy on Asphyxia, and on the best Means of restoring Suspended Animation .......... 234

7. Observations on Hydrophobia, original and selected .......... 236

8. Successful Amputation during the progress of Mortification in a Limb. By Dr. Heustis .......... 238

9. Curious Case of Scirrhous Testicle in the Groin .......... 239

10. Mr. Teale on Sulphate of Copper in Ophthalmia .......... 239

11. Curious Case of Fracture of a Cervical Vertebra by Muscular Contraction 240

ANALYTICAL REVIEW OF SELECT HOSPITAL REPORTS IN THE LANCET.

[BARTHOLOMEW'S HOSPITAL.]

1. Fatal Phlebitis .......... 241

2. Do. do. .......... 242

3. Nævus Maternus .......... 243

4. Dislocation, without Fracture, of Cervical Vertebræ .......... 243

5. Cancer of the Penis .......... 243

6. Erysipelas treated by Incisions .......... 244

7. Syphilis communicated to the Fœtus .......... 244

8. Phagedenic Ulceration .......... 245

9. Lepra Alphoides .......... 245

10. Impetigo Figurata .......... 245

11. Phlebitis .......... 245

[GUY'S HOSPITAL.]

1. Scrotal Hernia—unsuccessful Termination .......... 246

2. Fistula in Perineo .......... 247

3. Curious Case of Spinal Injury .......... 247

4. Fatal Spinal Disease .......... 247

5. Mr. Wardrop on Nævi Materni .......... 248

6. Spina Bifida cured .......... 248

EXTRA-LIMITES.

I.

PRIZE HOSPITAL REPORT, FROM THE WINCHESTER COUNTY HOSPITAL.
(Mr. BURY, Pupil.)

No. 2.

I. Intermittent Fevers, with Cases .......... 249
II. On Amputation, with Cases .......... 250
III. On Diseases of the Hip-joint .......... 254
1. Chronic Inflammation of the Synovial Membrane .......... 254
2. Apparent Elongation of the Thigh .......... 255
3. Shortening of the Thigh .......... 257
4. Ditto ditto .......... 257
IV. Secondary Symptoms of Syphilis .......... 259
1. Venereal Sore Throat treated by Mercury .......... 259
2. Syphilitic Sore Throat, with Inflammation of the Eyes, treated by Mercury .......... 260
3. Disease of the Bones of the Nose, after the excessive Use of Mercury .......... 260
V. Diseases of the Breast .......... 261
1. Encysted Tumour of the Breast .......... 261
2. Simple Chronic Tumour of the Breast .......... 262
3. Scirrhous Tubercle of the Breast .......... 262
VI. Case of Bronchocele successfully treated by Seton .......... 263
VII. Amputation of a finger, with its Metacarpal Bone .......... 264
VIII. Case of deep-seated Inflammation of the Eye .......... 265
IX. Case of Nasal Polypi .......... 265
X. Fracture of the Cervix Scapulæ .......... 266
XI. Operation of Lithotomy .......... 267
II. Dr. Harrison's Letter to Dr. Chambers .......... 268

ANALECTA MINORA.

1. Supposed Aneurism .......... 271
2. Anchylosis cured by an operation .......... 272
3. Hepatic Suppuration—Paracentesis Thoracis .......... 273
Remarkable Disease of the Liver .......... 274
4. Hernia, with Retention of Urine and Rupture of the Bladder .......... 275
5. Fracture of the Pelvis, with Rupture of the Urethra .......... 276
6. Aneurism at the Bend of the Arm .......... 277
7. Incontinence of Urine .......... 278
8. Ergot of Rye .......... 278
9. Unsuspected Abscess in the Liver .......... 278
10. Cas Rares—Remarkable Cases .......... 279
11. Acupuncture .......... 279
12. Nitrate of Potash in Menorrhagia .......... 279
13. Amaurosis .......... 279
14. Incisions in Erysipelas Phlegmonodes .......... 280
15. Noxious Exhalations .......... 280
16. Curious Case of Nævi Materni .......... 280
Bibliographical Record .......... 281

INTELLIGENCE, &c.

Clinical Assistants in Metropolitan Hospitals .......... 284
Incisions in Erysipelas .......... 285
Notices to Correspondents .......... 285
Internal Variolous Pustules .......... 285
Stomach Pump .......... 285
Mr. Cooke—Anatomical Persecution .......... 286
Medico-Chirurgical Society .......... 286
Emaciation of the Heart—Hypertrophy of the Heart .......... 287
List of Subscribers .......... 288

